# The Microglial Activation Inhibitor Minocycline, Used Alone and in Combination with Duloxetine, Attenuates Pain Caused by Oxaliplatin in Mice

**DOI:** 10.3390/molecules26123577

**Published:** 2021-06-11

**Authors:** Kinga Sałat, Anna Furgała-Wojas, Robert Sałat

**Affiliations:** 1 Department of Pharmacodynamics, Faculty of Pharmacy, Jagiellonian University Medical College, 9 Medyczna St., 30-688 Krakow, Poland; anna.furgala@student.uj.edu.pl; 2 Faculty of Electrical and Computer Engineering, Cracow University of Technology, 24 Warszawska St., 31-155 Krakow, Poland; robert.salat@pk.edu.pl

**Keywords:** oxaliplatin-induced neuropathic pain, minocycline, microglial inhibition, duloxetine, combination drug therapy

## Abstract

The antitumor drug, oxaliplatin, induces neuropathic pain, which is resistant to available analgesics, and novel mechanism-based therapies are being evaluated for this debilitating condition. Since activated microglia, impaired serotonergic and noradrenergic neurotransmission and overexpressed sodium channels are implicated in oxaliplatin-induced pain, this in vivo study assessed the effect of minocycline, a microglial activation inhibitor used alone or in combination with ambroxol, a sodium channel blocker, or duloxetine, a serotonin and noradrenaline reuptake inhibitor, on oxaliplatin-induced tactile allodynia and cold hyperalgesia. To induce neuropathic pain, a single dose (10 mg/kg) of intraperitoneal oxaliplatin was used. The mechanical and cold pain thresholds were assessed using mouse von Frey and cold plate tests, respectively. On the day of oxaliplatin administration, only duloxetine (30 mg/kg) and minocycline (100 mg/kg) used alone attenuated both tactile allodynia and cold hyperalgesia 1 h and 6 h after administration. Minocycline (50 mg/kg), duloxetine (10 mg/kg) and combined minocycline + duloxetine influenced only tactile allodynia. Seven days after oxaliplatin, tactile allodynia (but not cold hyperalgesia) was attenuated by minocycline (100 mg/kg), duloxetine (30 mg/kg) and combined minocycline and duloxetine. These results indicate a potential usefulness of minocycline used alone or combination with duloxetine in the treatment of oxaliplatin-induced pain.

## 1. Introduction

Chemotherapy-induced peripheral neuropathy (CIPN) is a major dose-limiting adverse effect of several chemotherapeutic agents, such as vincristine, taxanes, platinum derivatives (cisplatin, oxaliplatin), or bortezomib. Effective preventative and treatment options for CIPN and its main symptom, i.e., neuropathic pain, are limited. This clinical entity is often resistant to available analgesics, and is therefore regarded as a challenging and complex chronic disease with a major and prolonged impact on patients’ quality of life [1].

Oxaliplatin is a third-generation platinum-based derivative used for the treatment of colorectal cancer. CIPN induced by this drug is one of the most frequent and dose-limiting adverse effects. It is characterized by acute, transient neuropathy that occurs in almost 90% of patients within hours after infusion, as well as chronic peripheral neuropathy observed in 70% of oxaliplatin-treated patients. The symptoms of the chronic phase resemble those of the acute phase, although these delayed complications may persist for several months and can progressively worsen; this phenomenon is known as ‘coasting’ [2,3,4,5].

Acute cold and mechanical hypersensitivity (i.e., cold hyperalgesia and tactile allodynia) are noted in patients treated with oxaliplatin. The former is characterized by dysesthesias and paresthesias of the hands and feet, and these neurological symptoms are often induced or exacerbated by exposure to cold; motor symptoms might also appear. The acute symptoms of CIPN generally subside between the treatment cycles, although prolonged exposure to oxaliplatin can lead to the development of severe chronic neuropathy. This is thought to be a consequence of the accumulation of oxaliplatin in the dorsal root ganglia, which reduces neuronal metabolism and impairs axonal transport. Of note, since repeated episodes of acute neuronal hyperexcitability may lead to the structural damage of nerves, the chronic form of oxaliplatin-induced neurotoxicity is often irreversible [2,5]. Additionally, at high cumulative doses, alterations in proprioception might be observed [6].

Molecular mechanisms that contribute to the development of acute neuropathy in oxaliplatin-treated patients comprise various mechanisms, such as transient activation of voltage-gated ion (sodium (Na_v_), calcium (Ca_v_)) channels, impaired axonal transport, calcium homeostasis, microglial activation and increased oxidative stress in the peripheral nervous system, which results in the hyperexcitability of neuronal cell membranes [2,7,8,9,10,11,12]. Therefore, there is still a strong medical need to develop novel treatment options to not only relieve neuropathic pain in pre-existing CIPN caused by oxaliplatin, but also to develop effective, mechanism-based preventative therapies for CIPN [13]. For this reason, the prevention of the development of late-phase (irreversible) neuropathy is a subject of extensive pharmacological studies. At present, duloxetine, an antidepressant drug that inhibits serotonin and noradrenaline reuptake, is recommended by the American Society of Clinical Oncology for the treatment of established painful peripheral neuropathy and it is also being tested to prevent oxaliplatin-associated CIPN [1,6,13]. However, for the usefulness of duloxetine in CIPN-associated pain, moderate evidence has been gathered, with extrapolation from other neuropathic pain states and although this drug is used in patients on oxaliplatin therapy, the benefits from its use for this clinical indication still seem to be limited [14,15]. Apart from duloxetine, there is only one drug candidate, i.e., calmangafodipir, a mitochondrial enzyme manganese superoxide dismutase mimetic, currently undergoing a placebo-controlled, double-blinded randomized phase III study as a potential option to prevent the development of oxaliplatin-induced acute and delayed CIPN [16]. Although calmangafodipir appears to prevent the development of oxaliplatin-induced acute and delayed symptoms of CIPN, without an apparent influence on tumor progression and its protective effect was observed in the behavioral studies and confirmed by ex vivo evaluations, the data about its efficacy and safety are still preliminary [17].

In addition to the discovery of new chemical compounds and the development of novel drug candidates for CIPN, other approaches are being tested for their potential application in this clinical condition. Recently, it has been suggested that drug repurposing and combination drug therapy (CDT) might be useful approaches to the treatment of pain in the course of CIPN, as they may enable the expected pharmacotherapeutic effect to be achieved using drug doses that are ineffective in monotherapy [18,19,20,21]. Also, it is recommended to focus on CIPN mechanism-based therapies to attenuate its symptoms [9].

Considering the above-mentioned molecular mechanisms implicated in the development in CIPN, this present study performed in oxaliplatin-treated mice aimed to assess the antiallodynic and antihyperalgesic properties of minocycline, a semisynthetic tetracycline and a potent inhibitor of microglial activation with anti-inflammatory, antiapoptotic, and analgesic properties [22]. Recent studies have demonstrated the involvement of activated microglia and astrocytes in oxaliplatin-induced neuropathic pain [23]. It was also shown that the increased density of microglial cells and astrocytes correlates with pain hypersensitivity due to oxaliplatin administration [24], and decreased activation of the microglia and astrocytes relieved pain and stimulated neuroprotection [23,25]. Based on these findings [26,27,28,29,30,31,32,33], in this present research minocycline was used alone or in combination with a Na_v_ channel inhibitor, i.e., ambroxol [34,35,36], or a serotonin/noradrenaline reuptake inhibitor with Na_v_-channel blocking properties, duloxetine [37,38], to attenuate tactile allodynia and cold hyperalgesia caused by oxaliplatin. 

The present research aimed to assess whether drugs that influence the function of microglia, i.e., non-excitable cells expressing Na_v_ [39] and whose function is modulated by serotonin and noradrenaline [40], might be effective as treatment for the neuropathic pain in the course of CIPN caused by oxaliplatin.

## 2. Results

### 2.1. Experimental Design

In this in vivo research we assessed the effect of test substances, used alone or in combination, on the mechanical and thermal (cold) nociceptive threshold. Two control groups were used, namely, the vehicle-treated mice that were not given oxaliplatin, and the oxaliplatin-injected vehicle-treated mice. The use of these two control groups aimed to assess if oxaliplatin was able to reduce the pain threshold in mice. The effect of minocycline, duloxetine and ambroxol on the mechanical nociceptive threshold was assessed in oxaliplatin-exposed mice in the von Frey test, whereas their effect on the thermal nociceptive threshold was evaluated in the cold plate test.

The activities of the test drugs used alone (doses: minocycline—50 and 100 mg/kg; duloxetine—10 and 30 mg/kg) and in combinations (doses: minocycline 50 mg/kg, duloxetine 10 mg/kg, ambroxol 90 mg/kg) were compared to assess if protocols based on combined low-dose drugs were able to induce analgesic effects similar to those of high doses of drugs used alone.

Single-dose and repeated-dose protocols were used in this study. In the former one, test compounds were administered once on the day of oxaliplatin administration (an early observation time point) and then, also once daily, 7 days later (a late observation time point). The repeated-dose protocol was based on the administration of test drugs (minocycline, duloxetine, ambroxol and vehicle) for 7 consecutive days, once daily, starting on the day of oxaliplatin administration.

**Figure 1:**
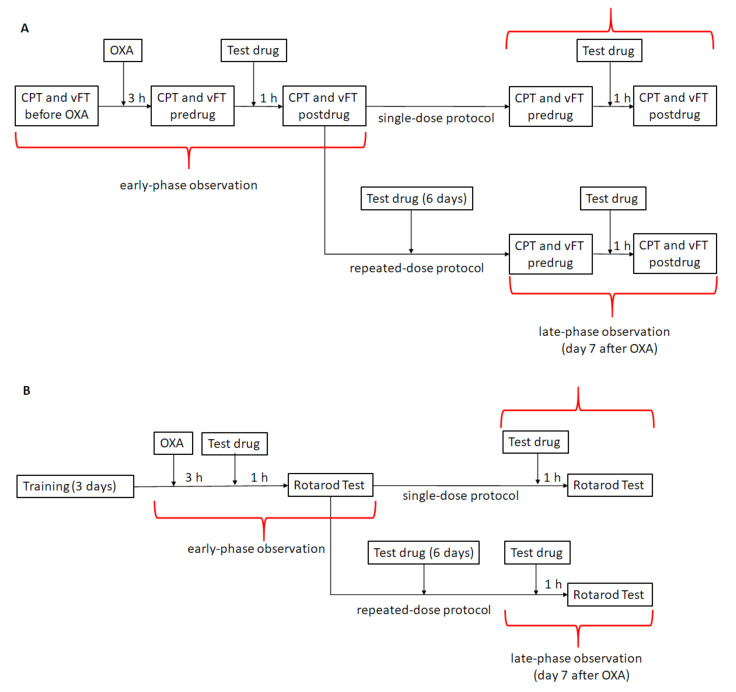
Schema showing drug administration and in vivo testing protocols used in the present study. (**A**) The intervention protocol used to measure the effects of single-dose or repeated-dose minocycline, ambroxol and duloxetine used alone or in combination on tactile allodynia in the von Frey test and cold hyperalgesia in the cold plate test in oxaliplatin-treated mice. (**B**) The protocol used for the measurement of the impact of these drugs on animals’ motor coordination assessed in the rotarod test. Abbreviations: CPT—cold plate test; vFT—von Frey test; OXA—oxaliplatin.

Similar protocols aiming to assess the effects of test drugs on motor coordination were used in the rotarod test. A detailed description of the doses of the drugs, the protocols of the drug administration and the route of the drug administration is shown in Figure 1 and described in Section 4.2 and Section 4.3.

### 2.2. Effect of Test Drugs Used Alone in a Single- or Repeated-Dose Protocol on Tactile Allodynia in Oxaliplatin-Treated Mice (Von Frey Test)

In the first stage of this research for minocycline and duloxetine used in monotherapy, the doses showing high and low antiallodynic activity in the von Frey test were established.

#### 2.2.1. Effect of Minocycline on Tactile Allodynia

**Figure 2:**
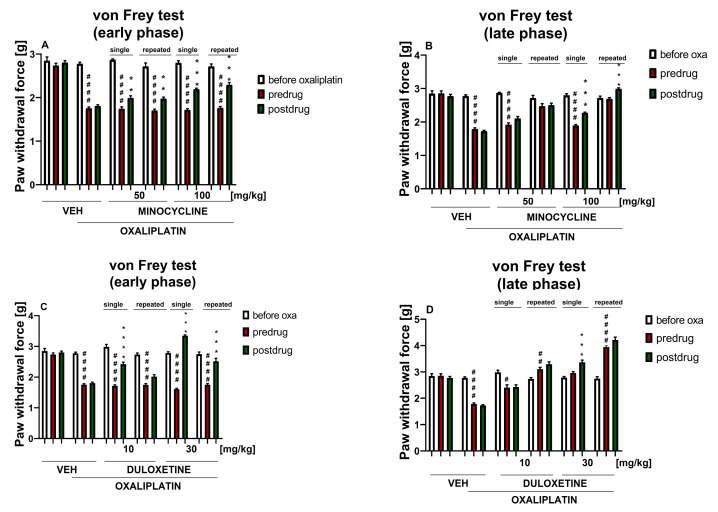
Effect of single-dose and repeated-dose minocycline and duloxetine, each used alone, on pain threshold in oxaliplatin-treated mice. Effect of minocycline (50, 100 mg/kg, i.p.) on tactile allodynia was measured in the mouse von Frey test on the day of oxaliplatin (oxa) administration (**A**) and 7 days later (**B**). Effect of duloxetine (10, 30 mg/kg, i.p.) on tactile allodynia was measured in the mouse von Frey test measured on the day of oxaliplatin administration (**C**) and 7 days later (**D**). The results are shown as the mean paw withdrawal threshold ± SEM for *n* = 9–10. Statistical analysis: repeated measures ANOVA followed by Tukey’s post-hoc comparison. Significance: # *p* < 0.05, ## *p* < 0.01, #### *p* < 0.0001 vs. before oxaliplatin; ** *p* < 0.01, *** *p* < 0.001, **** *p* < 0.0001 vs. predrug paw withdrawal threshold in the individual group. In the figure titles ‘early phase’ (**A**,**C**) and ‘late phase’ (**B**,**D**) mean two time points at which the measurements were taken (day of oxaliplatin administration and day 7 after a single-dose oxaliplatin administration, respectively). Numerical values of the paw withdrawal thresholds ± SEM presented in this figure are shown in the Supplementary Materials (Table S1).

Repeated measures ANOVA revealed an overall effect of the treatment (oxaliplatin, vehicle and minocycline) on the mechanical nociceptive threshold (F[5,54] = 117.3, *p* < 0.0001). Time affected the results significantly (F[3.341,180.4] = 284.5, *p* < 0.0001), and the drug—time interaction was also significant (F[20,216] = 30.84, *p* < 0.0001). Post-hoc analysis showed that, before oxaliplatin administration, the paw withdrawal threshold did not significantly differ among the groups. Oxaliplatin administration significantly lowered the mechanical nociceptive threshold (*p* < 0.0001 vs. vehicle-treated mice that did not receive oxaliplatin) and this lowered the pre-drug paw withdrawal threshold, which was an indicator of tactile allodynia noted in all of the oxaliplatin-treated groups in the von Frey test performed on the day of oxaliplatin administration (early stage of observation) (Figure 2A). A significantly (*p* < 0.0001) lowered (compared to the measurement before oxaliplatin) predrug paw withdrawal threshold was also noted 7 days after the oxaliplatin injection in the oxaliplatin-exposed groups, except for those repeatedly administered with minocycline 50 and 100 mg/kg (Figure 2B). This indicated that in oxaliplatin-treated mice, repeated (7 day) administrations of minocycline partially reduced tactile allodynia caused by oxaliplatin.

The comparison of pre- and post-drug paw withdrawal thresholds within each experimental group (Figure 2A,B) revealed significant differences in the oxaliplatin-treated mice that received single-dose and repeated-dose minocycline (100 mg/kg) (*p* < 0.001 at both time points of testing), and single-dose and repeated-dose minocycline (50 mg/kg) (*p* < 0.01 at the early observation time point). As shown in Figure 2B, in the oxaliplatin-treated mice that received single-dose minocycline (50 and 100 mg/kg) the pre-drug paw withdrawal thresholds on day 7 of testing were much lower than those of the mice repeatedly treated with minocycline. This finding confirms that the 7 day administration of minocycline (at both doses) attenuated tactile allodynia induced by oxaliplatin, almost restoring the mechanical nociceptive threshold of these repeated-dose minocycline-treated groups to values before oxaliplatin (and values of control mice not injected with oxaliplatin).

#### 2.2.2. Effect of Duloxetine on Tactile Allodynia

Repeated measures ANOVA revealed an overall effect of the treatment (oxaliplatin, duloxetine, vehicle) on the mechanical nociceptive threshold (F[5,54] = 131.1, *p* < 0.0001). Time affected the results significantly (F[3.183,171.9] = 297.2, *p* < 0.0001), and the drug–time interaction was also significant (F[20,216) = 67.41, *p* < 0.0001). Post-hoc analysis showed that, before oxaliplatin administration, the paw withdrawal threshold did not differ significantly among the groups. Oxaliplatin administration lowered the mechanical nociceptive threshold (*p* < 0.0001 vs. vehicle-treated mice that did not receive oxaliplatin) and this lowered the pre-drug paw withdrawal threshold, which was an indicator of tactile allodynia noted in all of the oxaliplatin-treated groups in the von Frey test performed on the day of oxaliplatin administration (early stage of observation) (Figure 2C).

A significantly lowered (compared to the measurement before oxaliplatin) pre-drug paw withdrawal threshold was also noted 7 days after oxaliplatin injection in the oxaliplatin-exposed control mice (*p* < 0.0001) and the single-dose duloxetine (10 mg/kg) group (*p* < 0.05). As shown in Figure 2D, at the late observation time point, the pre-drug paw withdrawal threshold in the single-dose duloxetine (30 mg/kg) group was similar to that before oxaliplatin, whereas in this late phase of the study, the pre-drug paw withdrawals in the repeated-dose duloxetine (10 and 30 mg/kg) groups were much higher as compared to the values before oxaliplatin. Taken together, this indicated that in oxaliplatin-treated mice, repeated (7 day) administrations of duloxetine reversed the tactile allodynia caused by oxaliplatin. Of note, this effect elevated far beyond the baseline values of healthy controls indicated for a potentially dangerous excessive pharmacological effect of repeatedly administered duloxetine, namely, a duloxetine-induced excessive increase in the mechanical nociceptive threshold in oxaliplatin-treated mice.

The comparison of pre- and post-drug paw withdrawal thresholds within each experimental group (Figure 2C,D) revealed significant differences in the oxaliplatin-treated mice that received single-dose duloxetine (10 mg/kg) only in the early phase of the study (*p* < 0.0001) and single-dose duloxetine (30 mg/kg) (*p* < 0.001) at both time points of testing. Repeated dosing of duloxetine (30 mg/kg) increased the mechanical nociceptive threshold, but it is noteworthy that this effect was significant only in the early phase of the present study. In this group of mice, differences between the pre-drug and post-drug paw withdrawal thresholds were not observed in the late phase of the study.

### 2.3. Effect of Test Drugs Used in Combination in a Single- or Repeated-Dose Protocol on Tactile Allodynia in Oxaliplatin-Treated Mice (Von Frey Test)

In the next stage of our experiment, we aimed to assess whether the addition of ambroxol or duloxetine at doses that showed low activity in pain tests when used in monotherapy (90 and 10 mg/kg, respectively) might enhance the antiallodynic properties of low-dose minocycline (50 mg/kg).

#### 2.3.1. Effect of Minocycline Combined with Ambroxol on Tactile Allodynia

In our recent study [41], we demonstrated that intraperitoneal ambroxol (90 mg/kg) used as a single or repeated dose attenuated tactile allodynia in oxaliplatin-treated mice. In the von Frey test, significant differences were noted between the pre-drug paw withdrawal threshold in the early phase of the study and the pre-drug paw withdrawal threshold in the late phase of the study (day 7 after oxaliplatin) for repeated-dose ambroxol (90 mg/kg). This finding indicated that the repeated administration of this drug partially attenuated the tactile allodynia caused by oxaliplatin [41].

In the present experiment, repeated measures ANOVA revealed an overall effect of treatment (oxaliplatin, minocycline, ambroxol and vehicle) on the mechanical nociceptive threshold (F[7,72] = 65.52, *p* < 0.0001). Time significantly affected the results (F[3.652,262.9] = 411.9, *p* < 0.0001), and the drug–time interaction was also significant (F[28,288) = 23.69, *p* < 0.0001). Post-hoc analysis showed that, before oxaliplatin administration, the paw withdrawal threshold did not differ significantly among the groups. Oxaliplatin administration lowered the nociceptive threshold for mechanical stimulation (*p* < 0.0001 vs. vehicle-treated mice that did not receive oxaliplatin).

**Figure 3:**
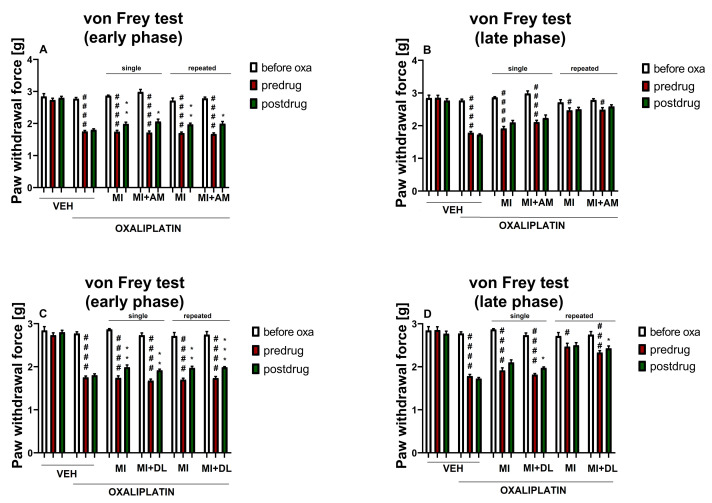
Effect of single-dose and repeated-dose minocycline combined with ambroxol or duloxetine on pain threshold in oxaliplatin-treated mice. Effect of minocycline (50 mg/kg, i.p.) combined with ambroxol (90 mg/kg, i.p.) measured in the von Frey test on the day of oxaliplatin administration (**A**) and 7 days later (**B**). Effect of minocycline (50 mg/kg, i.p.) combined with duloxetine (10 mg/kg, i.p.) on tactile allodynia measured in the von Frey test on the day of oxaliplatin administration (**C**) and 7 days later (**D**). The results for minocycline used in combination and minocycline used alone (50 mg/kg) are shown as the mean paw withdrawal threshold ± SEM for *n* = 9–10. Statistical analysis: repeated measures ANOVA followed by Tukey’s post-hoc comparison. Significance: # *p* < 0.05, ### *p* < 0.001, #### *p* < 0.0001 vs. before oxaliplatin; * *p* < 0.05, ** *p* < 0.01, *** *p* < 0.001 vs. pre-drug paw withdrawal threshold in the individual group. Abbreviations: oxa—oxaliplatin, MI—minocycline, AM—ambroxol, DL—duloxetine. In the figure titles ‘early phase’ (**A**,**C**) and ‘late phase’ (**B**,**D**) mean two time points at which the measurements were taken (day of oxaliplatin administration and day 7 after a single-dose oxaliplatin administration, respectively). Numerical values of the paw withdrawal thresholds ± SEM presented in this figure are shown in the Supplementary Materials (Table S1).

In the early phase of this study, in the oxaliplatin-treated mice, single-dose and repeated-dose minocycline (50 mg/kg) + ambroxol (90 mg/kg) were almost equally as effective as single-dose minocycline (100 mg/kg). Additionally, in this phase, single-dose and repeated-dose minocycline (50 mg/kg) + ambroxol (90 mg/kg) were almost equally as effective as repeated-dose minocycline (100 mg/kg) (Figure 3A vs. Figure 2A). At this observation time point, the addition of ambroxol (90 mg/kg) to minocycline (50 mg/kg) (single- and repeated-dose protocols) was not superior to single-dose or repeated-dose minocycline (50 mg/kg) used alone (Figure 3A).

Significant differences in the pre-drug paw withdrawal threshold in the late phase of this study were noted between the control mice that did not receive oxaliplatin and the combined single-dose minocycline (50 mg/kg) + ambroxol (90 mg/kg) group (*p* < 0.0001). Also, such differences were observed between the control mice not injected with oxaliplatin and the repeated-dose minocycline (50 mg/kg) + ambroxol (90 mg/kg) group (*p* < 0.05). At this late time point of observation, the pre-drug paw withdrawal threshold in the oxaliplatin-treated mice that received repeated-dose minocycline (100 mg/kg) was increased compared to that of the oxaliplatin-treated mice that received single-dose minocycline (50 mg/kg) + ambroxol (90 mg/kg) (*p* < 0.0001), but similar to that of the mice that received repeated-dose minocycline (50 mg/kg) + ambroxol (90 mg/kg) (Figure 2B vs. Figure 3B).

Single-dose minocycline (50 mg/kg) + ambroxol (90 mg/kg) was equally effective to single-dose minocycline (50 mg/kg) alone (Figure 3A,B). Repeated dosing of minocycline (50 mg/kg) used alone had a similar effect on the late-phase pre-drug paw withdrawal threshold as repeated-dose minocycline (50 mg/kg) + ambroxol (90 mg/kg), but was more effective than the single-dose drug combination (*p* < 0.05; Figure 3B).

Similar to the results obtained for minocycline (50 mg/kg) alone, the comparison of the pre-drug and post-drug paw withdrawal thresholds in the combined minocycline (50 mg/kg) and ambroxol (90 mg/kg)-treated groups revealed a significant antiallodynic effect of a single-dose and repeated-dose treatment in the early phase of this study (*p* < 0.05), but not in the late phase (Figure 3A,B).

Taken together, this part of the study did not demonstrate significant differences in the antiallodynic action between minocycline used alone and in combination with ambroxol. Therefore, it can be concluded that the potential clinical usefulness of such a drug combination is marginal.

#### 2.3.2. Effect of Minocycline Combined with Duloxetine on Tactile Allodynia

Repeated measures ANOVA revealed an overall effect of treatment (oxaliplatin, minocycline, duloxetine and vehicle), (F[7,72] = 107.5, *p* < 0.0001). The time effect and the drug–time interaction were also significant (F[3.352, 241.4] = 442.4, *p* < 0.0001, and F[28,288] = 26.02, *p* < 0.0001, respectively).

The injection of oxaliplatin significantly lowered the pain threshold of mice (*p* < 0.0001 vs. measurements before oxaliplatin). On the day of oxaliplatin administration, the pre-drug paw withdrawal threshold of the mice treated with combined minocycline and duloxetine (and oxaliplatin) was similar to that of all of the other oxaliplatin-treated groups. In this phase of the study, single-dose combined minocycline and duloxetine increased the paw withdrawal threshold less effectively than single-dose and repeated-dose minocycline (100 mg/kg) used alone (*p* < 0.001), and was equally as effective as single-dose and repeated-dose minocycline (50 mg/kg) used alone. Repeated-dose combined minocycline and duloxetine was less effective than single-dose and repeated-dose minocycline (100 mg/kg) (*p* < 0.01), and was equally as effective as minocycline (50 mg/kg) alone (Figure 3C vs. Figure 2A).

The late-phase pre-drug paw withdrawal threshold was significantly lower (*p* < 0.001) in the single-dose and repeated-dose combined minocycline + duloxetine (and oxaliplatin)-treated group than in the control mice not treated with oxaliplatin.

As shown in Figure 3D, on day 7 of this study, the combined administration of single-dose minocycline and duloxetine attenuated tactile allodynia in oxaliplatin-treated mice less effectively than the repeated dosing of this drug combination (*p* < 0.0001).

In the late phase of this study, in the repeated-dose combined minocycline + duloxetine group, the pre-drug paw withdrawal threshold was significantly lower as compared to the measurements before oxaliplatin. It was also lower than that of the repeated-dose minocycline (100 mg/kg) (*p* < 0.001) group, but higher than that of the oxaliplatin-treated control, single-dose minocycline (100 mg/kg) and single-dose combined minocycline and duloxetine (*p* < 0.001) (Figure 3D vs. Figure 2B).

The comparison of the pre- and post-drug paw withdrawal thresholds in the combined minocycline + duloxetine-treated groups showed that, in contrast to single-dose minocycline (50 mg/kg), which was active only on the day of oxaliplatin administration, the combined single-dose treatment showed antiallodynic properties in both the early phase (*p* < 0.01) and the late phase of this study (*p* < 0.05; Figure 3C,D vs. Figure 2A,B). Similarly, although repeated-dose minocycline (50 mg/kg) was able to increase the paw withdrawal threshold only in the early phase of the observation, the combined repeated-dose treatment showed antiallodynic properties in both the early phase (*p* < 0.001) and the late phase of this study (*p* < 0.05; Figure 3C,D vs. Figure 2A,B).

Taken together, this part of the study demonstrated that the use of combined minocycline and duloxetine was effective in both the early and the late phase of the observation, whereas minocycline at the same dose and used alone showed antiallodynic activity only on the day of oxaliplatin administration (Figure 3C,D). Therefore, it can be concluded that the potential usefulness of such a drug combination might be clinically important.

### 2.4. Duration of the Effect on Tactile Allodynia

**Figure 4:**
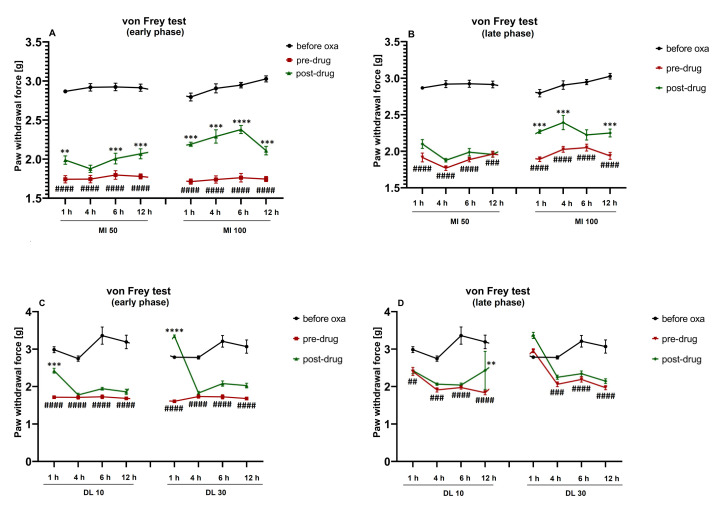
Duration of the antiallodynic effect of single-dose minocycline and duloxetine, each used alone, on the pain threshold in oxaliplatin-treated mice. Effect of minocycline (50, 100 mg/kg, i.p.) on tactile allodynia measured in the von Frey test assessed 1 h, 4 h, 6 h and 12 h after administration, on the day of oxaliplatin administration (**A**) and 7 days after oxaliplatin (**B**). Effect of duloxetine (10, 30 mg/kg, i.p.) on tactile allodynia measured in the von Frey test assessed 1 h, 4 h, 6 h and 12 h after administration, on the day of oxaliplatin administration (**C**) and 7 days after oxaliplatin (**D**). The results are shown as the mean paw withdrawal threshold ± SEM for *n* = 10. Statistical analysis: repeated measures ANOVA followed by Tukey’s post-hoc comparison. Significance: ## *p* < 0.01, ### *p* < 0.001, #### *p* < 0.0001 vs. before oxaliplatin; ** *p* < 0.01, *** *p* < 0.001, **** *p* < 0.0001 vs. pre-drug paw withdrawal threshold. Abbreviations: MI—minocycline, DL—duloxetine. In the figure titles ‘early phase’ (**A**,**C**) and ‘late phase’ (**B**,**D**) mean two time points at which the measurements were taken (day of oxaliplatin administration and day 7 after a single-dose oxaliplatin administration, respectively). Numerical values of the paw withdrawal thresholds ± SEM presented in this figure are shown in the Supplementary Materials (Table S2).

We also investigated the duration of the antiallodynic effect exerted by minocycline (Figure 4A,B) and duloxetine (Figure 4C,D), each used alone, in oxaliplatin-treated mice.

Repeated measures ANOVA showed an overall effect of treatment with oxaliplatin and minocycline (F[7,360] = 13.30, *p* < 0.0001). The time effect and drug–time interaction were also significant (F[4,360] = 636.3, p < 0.0001 and F[28,360] = 4.022, *p* < 0.0001, respectively). Post-hoc analysis revealed that, in the oxaliplatin-treated mice, minocycline (50 mg/kg) 1 h, 6 h and 12 h after its administration significantly (*p* < 0.01 vs. pre-drug paw withdrawal threshold) reduced tactile allodynia, but this effect was noted only in the early phase of the observation (Figure 4A). In contrast, the 100 mg/kg minocycline showed antiallodynic properties at both time points of the observation (*p* < 0.001) when tested 1 h, 4 h and 12 h after its administration (Figure 4A,B). The assessment of the antiallodynic effect of 100 mg/kg minocycline in oxaliplatin-treated mice measured in the von Frey test 6 h later showed its antiallodynic properties only in the early phase of the observation (*p* < 0.0001 vs. pre-drug paw withdrawal threshold; Figure 4A).

Repeated measures ANOVA showed an overall effect of the treatment in oxaliplatin- and duloxetine-treated mice (F[7,360] = 21.98, *p* < 0.0001). The time effect and the drug–time interaction were also significant (F[4,360] = 142.4, p < 0.0001; F[28,360] = 6.932, *p* < 0.0001, respectively). The antiallodynic effect of duloxetine (10 and 30 mg/kg) measured in the von Frey test in the oxaliplatin-treated mice was mainly noted 1 h after its administration and was limited to the early phase of the observation (Figure 4C).

### 2.5. Influence of Test Drugs Used Alone in a Single- or Repeated-Dose Protocols on Cold Hyperalgesia in Oxaliplatin-Treated Mice (Cold Plate Test)

In this part of the present research we assessed if minocycline and duloxetine, each used alone, in a single-dose or a repeated-dose protocol, were able to attenuate cold hyperalgesia caused by oxaliplatin. The doses of the drugs tested in the present experiment were the same as those used in the von Frey test.

#### 2.5.1. Effect of Minocycline on Cold Hyperalgesia

**Figure 5:**
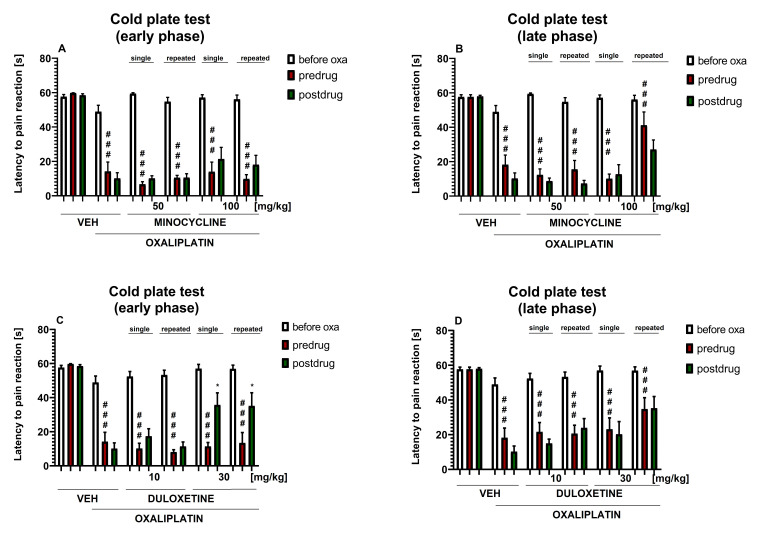
Effect of single-dose and repeated-dose minocycline and duloxetine, each used alone, on pain threshold in oxaliplatin-treated mice. Effect of minocycline (50, 100 mg/kg, i.p.) on cold hyperalgesia was measured in the cold plate test in mice on the day of oxaliplatin (oxa) administration (**A**) and 7 days later (**B**). Effect of duloxetine (10, 30 mg/kg, i.p.) on cold hyperalgesia was measured in the cold plate test in mice on the day of oxaliplatin administration (**C**) and 7 days later (**D**). The results are shown as the mean latency to pain reaction [s] ± SEM for *n* = 9–10. Statistical analysis: repeated measures ANOVA followed by Tukey’s post-hoc comparison. Significance: ### *p* < 0.001 vs. before oxaliplatin; * *p* < 0.05 vs. pre-drug latency to pain reaction in the individual group. In the figure titles ‘early phase’ (**A**,**C**) and ‘late phase’ (**B**,**D**) mean two time points at which the measurements were taken (day of oxaliplatin administration and day 7 after a single-dose oxaliplatin administration, respectively). Numerical values of the latencies to pain reaction ± SEM presented in this figure are shown in the Supplementary Materials (Table S3).

Repeated measures ANOVA revealed an overall effect of the treatment (oxaliplatin, minocycline and vehicle) on the cold nociceptive threshold (F[5,54] = 40.84, *p* < 0.0001). Time also significantly affected the results (F[3.332, 179.9] = 137.2, *p* < 0.0001), and the drug–time interaction was also significant (F[20,216] = 9.099, *p* < 0.0001). Post-hoc analysis showed that, although the latencies to pain reaction before oxaliplatin treatment were similar in all of the experimental groups, a statistically significant difference in the pre-drug latency to pain reaction was noted between the oxaliplatin-treated mice and the control mice not treated with oxaliplatin (*p* < 0.001). This difference was noted at both time points of testing (Figure 5A,B) and it indicated that, in mice, oxaliplatin induced cold hyperalgesia.

**Figure 6:**
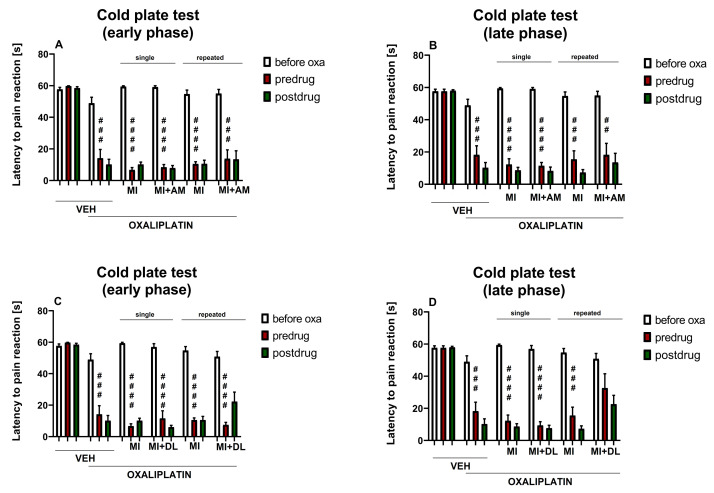
Effect of single-dose and repeated-dose minocycline combined with ambroxol or duloxetine on pain threshold in oxaliplatin-treated mice. Effect of minocycline (50 mg/kg, i.p.) combined with ambroxol (90 mg/kg, i.p.) on cold hyperalgesia measured in the cold plate test on the day of oxaliplatin administration (**A**) and 7 days later (**B**). Effect of minocycline (50 mg/kg, i.p.) combined with duloxetine (10 mg/kg, i.p.) on cold hyperalgesia measured in the cold plate test on the day of oxaliplatin administration (**C**) and 7 days later (**D**). The results for minocycline used in combination and minocycline used alone (50 mg/kg) are shown as the mean latency to pain reaction ± SEM for *n* = 9–10. Statistical analysis: repeated measures ANOVA followed by Tukey’s post-hoc comparison. Significance: ## *p* < 0.01, ### *p* < 0.001, #### *p* < 0.0001 vs. before oxaliplatin. Abbreviations: oxa—oxaliplatin, MI—minocycline, AM—ambroxol, DL—duloxetine. In the figure titles ‘early phase’ (**A**,**C**) and ‘late phase’ (**B**,**D**) mean two time points at which the measurements were taken (day of oxaliplatin administration and day 7 after a single-dose oxaliplatin administration, respectively). Numerical values of the latencies to pain reaction ± SEM presented in this figure are shown in the Supplementary Materials (Table S3).

**Figure 7:**
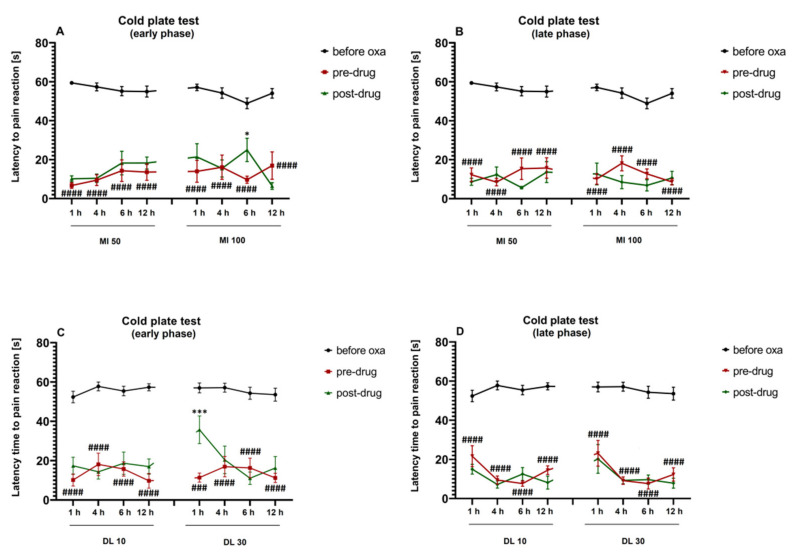
Duration of the antihyperalgesic effect of single-dose minocycline and duloxetine, each used alone, on the pain threshold in oxaliplatin-treated mice. Effect of minocycline (50, 100 mg/kg, i.p.) on cold hyperalgesia measured in the cold plate test assessed 1 h, 4 h, 6 h and 12 h after administration, on the day of oxaliplatin administration (**A**) and 7 days later (**B**). Effect of duloxetine (10, 30 mg/kg, i.p.) on cold hyperalgesia measured in the cold plate test assessed 1 h, 4 h, 6 h and 12 h after administration, on the day of oxaliplatin administration (**C**) and 7 days later (**D**). The results are shown as the mean latency to pain reaction ± SEM for *n* = 10. Statistical analysis: repeated measures ANOVA followed by Tukey’s post-hoc comparison. Significance: ### *p* < 0.001, #### *p* < 0.0001 vs. latency before oxaliplatin; * *p* < 0.05, *** *p* < 0.001 vs. pre-drug latency to pain reaction. Abbreviations: MI—minocycline, DL—duloxetine. In the figure titles ‘early phase’ (**A**,**C**) and ‘late phase’ (**B**,**D**) mean two time points at which the measurements were taken (day of oxaliplatin administration and day 7 after a single-dose oxaliplatin administration, respectively). Numerical values of the latencies to pain reaction ± SEM presented in this figure are shown in the Supplementary Materials (Table S4).

The comparison of pre-and post-drug latencies to pain reaction, measured in the cold plate in each individual group, showed that neither single-dose nor repeated-dose minocycline (50 and 100 mg/kg) was able to increase the cold pain threshold in the oxaliplatin-treated mice, either in the early or the late phase of the observation (Figure 5A,B). Of note, 7 days after oxaliplatin a decrease in the post-drug latency to pain reaction compared to pre-drug latency was observed. Importantly, this effect (also observed for other drugs tested in this study—please see Figure 5D, Figure 6 and Figure 7) might be regarded as a limitation of this research. A potential explanation for this finding is that a confounding factor in behavioral pain tests, such as the hot and cold plate tests, is the tendency of mice for learned behavioral responses, which leads to reduced reaction times during subsequent testing sessions [42].

Of note, although the early-phase pre-drug latencies were similar in both the repeated-dosing and the single-dosing protocols of the 100 mg/kg minocycline, the repeated dosing of the 100 mg/kg minocycline significantly (compared to single-dosing) prolonged the late-phase pre-drug latency to pain reaction. This finding might indicate that repeated administrations of minocycline at a dose of 100 mg/kg partially attenuated the development of late-phase cold hyperalgesia measured in the oxaliplatin-treated mice 7 days after oxaliplatin administration (Figure 5A,B).

#### 2.5.2. Effect of Duloxetine on Cold Hyperalgesia

Repeated measures ANOVA revealed an overall effect of the treatment (oxaliplatin, duloxetine and vehicle) on the cold nociceptive threshold (F[5,53] = 26.54, *p* < 0.0001). Time also affected the results significantly (F[3.586, 19.0] = 73.61, *p* < 0.0001), and the drug–time interaction was also significant (F[20,212] = 5.134, *p* < 0.0001). Post-hoc analysis showed that, although the latencies to pain reaction before oxaliplatin treatment were similar in all of the experimental groups, a statistically significant difference in the pre-drug latency to pain reaction was noted between the oxaliplatin-treated mice and the control mice not treated with this antitumor drug (*p* < 0.001). This difference was observed on the day of oxaliplatin administration and 7 days later (Figure 5C,D).

The comparison of the pre-and post-drug latencies to pain reaction, measured in the cold plate test, revealed that neither single-dose nor repeated-dose duloxetine (10 mg/kg) prolonged the latency to pain reaction in the early phase or the late phase of the study. In contrast, the single and repeated doses of 30 mg/kg duloxetine attenuated early-phase cold hyperalgesia in the oxaliplatin-treated mice (*p* < 0.05; Figure 5C).

Of note, although the early-phase pre-drug latencies were similar in both the repeated-dosing and the single-dosing protocols of duloxetine (30 mg/kg) (Figure 5C), the repeated dosing of duloxetine (30 mg/kg) significantly (*p* < 0.05 compared to single-dosing) prolonged the late-phase pre-drug latency to pain reaction. This finding might indicate that repeated administrations of duloxetine, at a dose of 30 mg/kg, partially attenuated the development of cold hyperalgesia measured in the oxaliplatin-treated mice 7 days after oxaliplatin (Figure 5D).

### 2.6. Influence of Test Drugs Used in Combination in a Single- or Repeated-Dose Protocol on Cold Hyperalgesia in Oxaliplatin-Treated Mice (Cold Plate Test)

In the next stage of our experiment, we aimed to assess whether the addition of ambroxol or duloxetine, at doses that showed low activity in pain tests when used in monotherapy (90 and 10 mg/kg, respectively), might enhance the antihyperalgesic properties of low-dose minocycline (50 mg/kg) assessed in the cold plate test.

#### 2.6.1. Effect of Minocycline Combined with Ambroxol on Cold Hyperalgesia

Our previous study showed that intraperitoneal ambroxol (90 mg/kg) used alone did not increase the cold pain threshold of oxaliplatin-treated mice [41].

In the present study, repeated measures ANOVA revealed an overall effect of the treatment (oxaliplatin, minocycline, ambroxol and vehicle) (F[7,72] = 34.29, *p* < 0.0001). Time and the drug–time interaction were also significant (F[3.354, 241.5] = 187.5, *p* < 0.0001 and F[28,288] = 6.329, *p* < 0.0001, respectively). Post-hoc analysis revealed that in the cold plate test in the oxaliplatin-treated mice, single-dose or repeated-dose combined minocycline (50 mg/kg) and ambroxol (90 mg/kg), similar to minocycline (50 and 100 mg/kg) used alone, were not effective in reducing cold hyperalgesia (Figure 6A,B). Based on the results obtained, it can be concluded that such a drug combination will not be clinically useful.

#### 2.6.2. Effect of Minocycline Combined with Duloxetine on Cold Hyperalgesia

Repeated measures ANOVA revealed an overall effect of the treatment (oxaliplatin, minocycline, duloxetine and vehicle) (F[7,71] = 30.39, *p* < 0.0001). Time and the drug–time interaction were also significant (F[3.296, 234.0] = 180.9, *p* < 0.0001 and F[28,284] = 7.230, *p* < 0.0001, respectively).

On the day of oxaliplatin administration, combined minocycline (50 mg/kg) and duloxetine (10 mg/kg) were not effective in increasing the cold pain threshold lowered by oxaliplatin (Figure 6C). On day 7 after oxaliplatin, repeated dosing of this drug combination prolonged the pre-drug latency to pain reaction, and in these mice, the latency to pain reaction was not significantly different from that of the controls not treated with oxaliplatin (Figure 6D). Of note, this beneficial effect was not observed for repeated-dose minocycline (50 mg/kg) or duloxetine (10 mg/kg) when used alone (Figure 5B,D).

The comparison of pre- and post-drug cold plate test latencies to pain reaction measured in the combined minocycline + duloxetine-treated groups showed that minocycline and duloxetine used in combination were not able to attenuate cold hyperalgesia in the early phase of this study (Figure 6C) or in the late one (Figure 6D).

Taken together, this part of our research did not demonstrate significant differences in the antihyperalgesic action between minocycline (50 mg/kg) used alone or in combination with duloxetine (10 mg/kg). Therefore, it can be concluded that the potential clinical usefulness of such a drug combination in reducing cold hyperalgesia is rather implausible.

### 2.7. Duration of the Effect on Cold Hyperalgesia

We also investigated the duration of the antihyperalgesic effect exerted by minocycline (Figure 7A,B) and duloxetine (Figure 7C,D), each used alone, in oxaliplatin-treated mice.

Repeated measures ANOVA did not reveal an overall effect of the treatment with oxaliplatin and minocycline (F[7,360] = 0.9120, *p* > 0.05). The time effect was significant (F[4,360] = 195.9, *p* < 0.0001), but the drug–time interaction was not (F[28,360] = 1.166, *p* > 0.05). Post-hoc analysis showed that the 50 mg/kg minocycline did not attenuate cold hyperalgesia 1 h, 4 h, 6 h or 12 h after injection in the early phase or the late phase of the observation (Figure 7A,B). In contrast, 100 mg/kg minocycline showed antihyperalgesic properties in the cold plate test, in the early phase of this study, 6 h after injection (*p* < 0.05 vs. pre-drug latency to pain reaction; Figure 7A).

Repeated measures ANOVA showed an overall effect of the treatment with oxaliplatin and duloxetine (F[7,360] = 3.009, *p* < 0.01). The time effect was significant (F[4,360] = 182.0, *p* < 0.0001), but the drug–time interaction was not (F[28,360] = 1.322, *p* > 0.05). Post-hoc analysis showed that duloxetine (10 mg/kg) did not attenuate oxaliplatin-induced cold hyperalgesia 1 h, 4 h, 6 h or 12 h after injection in the early phase or the late phase of the observation (Figure 7C,D). In contrast, the 30 mg/kg duloxetine showed antihyperalgesic properties in the cold plate test during the early phase of the study, 1 h after injection (*p* < 0.001 vs. pre-drug latency to pain reaction; Figure 7C).

Taken together, we demonstrated that in the early phase of the observation, the 100 mg/kg minocycline was able to reduce both tactile allodynia and cold hyperalgesia 6 h after administration. Duloxetine (30 mg/kg) attenuated both of the key painful symptoms caused by oxaliplatin 1 h after intraperitoneal administration (Figure 4 and Figure 7).

### 2.8. Influence on Motor Coordination-Rotarod Test

In the rotarod test, the effect on the motor coordination of the oxaliplatin-treated mice of test drugs administered in a single-dose or a repeated-dose protocol, and used alone or in combination, was assessed (Table 1). Oxaliplatin did not influence the animals’ motor coordination, either at 6, 18 or 24 rpm.

At 6 rpm, an overall effect of the treatment (oxaliplatin, minocycline, duloxetine, ambroxol and vehicle) was observed (F[19,136] = 2.014, *p* < 0.05). Post-hoc analysis revealed that at 6 rpm, no motor deficits were observed in the drug-treated groups compared to the oxaliplatin-treated control. At 18 rpm, one-way ANOVA revealed a statistically significant effect of the treatment (F[19,136] = 7.368, *p* < 0.0001). Post-hoc comparison revealed that single-dose duloxetine (10 mg/kg) significantly impaired the motor coordination of the oxaliplatin-treated mice during the late phase of the observation (*p* < 0.0001 vs. oxaliplatin-treated control mice). A similar motor-impairing effect was noted in the single-dose duloxetine (30 mg/kg)-treated mice during the early phase of the observation (*p* < 0.01 vs. oxaliplatin-treated control).

At 24 rpm, a significant effect of the treatment on the animals’ motor coordination was noted (F[19,136] = 8.480, *p* < 0.0001). Post-hoc analysis demonstrated that single-dose duloxetine (10 mg/kg) significantly impaired the motor coordination of the oxaliplatin-treated mice during the late phase of the present study (*p* < 0.0001 vs. oxaliplatin-treated control), while single-dose and repeated-dose duloxetine (30 mg/kg) induced severe motor deficits in the oxaliplatin-treated neuropathic mice at both time points of testing (*p* < 0.01 vs. oxaliplatin-treated control).

Taken together, the results of the present research showed that the low dose of duloxetine induced motor deficits similar to those caused by the high dose of this drug. These motor deficits might be, at least in part, responsible for the above-mentioned (in Section 2.2.2) excessive pharmacological effect of duloxetine noted in the von Frey test in oxaliplatin-treated mice.

Of note, motor-impairing properties caused by the dose of 10 mg/kg appeared later than those of the dose of 30 mg/kg, and they were not seen after repeated dosing. In contrast, the dose of 30 mg/kg induced motor deficits in the early phase of the observation and these deficits were maintained until the late phase of this study. These impairments of motor coordination, induced by duloxetine (30 mg/kg), were at a similar range as those reported in our previous research for the γ-aminobutyric acid transporter inhibitor, namely, tiagabine, also used at the dose of 30 mg/kg [43].

Minocycline alone did not impair the motor coordination of the oxaliplatin-treated mice. Moreover, as shown in Table 1, when used in combination with duloxetine (10 mg/kg) it was able to attenuate motor impairments caused by duloxetine.

## 3. Discussion

The results of the present research have shown that single and repeated administrations of the glial cell inhibitor minocycline, used alone or in combination with ambroxol or duloxetine, might attenuate pain due to an oxaliplatin injection. Minocycline has a long-lasting antiallodynic effect and, in mice, combined low-dose minocycline and duloxetine alleviated the neuropathic pain caused by oxaliplatin more effectively than each of these drugs used at the same doses in monotherapy.

Acute cold and mechanical hypersensitivity (i.e., cold hyperalgesia and tactile allodynia, respectively) that develops within hours after oxaliplatin administration, and its delayed, chronic form, are observed both in humans and in rodents. These two forms of CIPN depend on distinct molecular mechanisms, and in rodents they can be modeled by measuring both early (acute) and delayed responses to a single dose of intraperitoneally administered oxaliplatin. In mice, these early reactions appear 2–3 h after oxaliplatin administration, while subacute responses occur several days later. It has been shown that the hypersensitivity to painful stimuli detected 2 h after oxaliplatin administration might be different from those observed 24 h to 7 days after a single dose of oxaliplatin. Moreover, cold allodynia and cold hyperalgesia occur earlier than mechanical allodynia [41]. Considering these findings, in order to gain a full insight into the effects of test drugs on the painful reaction caused by oxaliplatin, we carried out the present experiment at two time points to distinguish the effects of the investigated drugs on acute and subacute responses of mice to oxaliplatin.

In our present research, we used the interventional protocol of test drug administration, i.e., we assessed the antiallodynic and antihyperalgesic properties of minocycline, ambroxol and duloxetine in pre-existing neuropathy caused by a single-dose oxaliplatin injection. However, it should be noted that we focused not only on the antiallodynic and antihyperalgesic properties of the test drugs used interventionally (i.e., in each phase of this study, we compared whether the test drugs are able to increase the nociceptive threshold for mechanical or thermal stimulation by comparing the pre-drug and post-drug readouts), but also aimed to establish whether single-dose or repeated-dose treatments were able to prevent the development of late-phase tactile allodynia and cold hyperalgesia in oxaliplatin-treated mice. This part of our study was particularly important in view of the previously mentioned persistence and drug resistance of the late-phase symptoms of neuropathy caused by oxaliplatin [1,44].

In the present research, we focused on drugs with distinct mechanisms of action and it should be noted that, for these three drugs, the molecular targets are located in many tissue types, including the nervous system, neurons and the glial tissue [39,40,45].

In the von Frey test, we showed that single-dose and repeated-dose minocycline (50 mg/kg) used in monotherapy was effective in reducing tactile allodynia in oxaliplatin-treated mice only in the early phase of the study. In contrast, the use of minocycline in the same protocols, but at a two-fold higher dose, was effective at both time points of testing (Figure 2A,B). This result confirms that the mechanisms regulating the mechanical nociceptive threshold during the acute-phase observation might be distinct from those that contribute to the late-phase one [46], and that the pharmacological effect of minocycline is dose-dependent, as shown previously in other neuropathic pain models [47,48].

Spinal and brain microglia play an important role in various neuropathic pain states [49,50,51,52,53,54]. Previously, it was demonstrated that the expression of astrocytes and microglia was increased in the anterior cingulate cortex of nerve-ligated mice. This effect was reversed by intracerebroventricularly administered minocycline, and it was suggested that the activation of microglia in the anterior cingulate cortex was involved in the development of hyperalgesia in mice with neuropathic pain [55]. The activation of microglia was also shown in fibromyalgia [56], diabetic neuropathic pain [57,58] and CIPN caused by vincristine [59] and cisplatin [60]. Hence, it was suggested that the inhibition of activated microglia might be a potential therapeutic strategy for the attenuation of neuropathic pain [61].

Available data indicate that minocycline shows a broad spectrum of mechanisms [45] that result in antinociceptive, antiallodynic and antihyperalgesic properties of this drug in a variety of pain models [53,56,62], including neuropathic pain models induced by traumatic nerve injury [49,63], chemotherapy, diabetes or spinal cord injury. Minocycline was also found to be effective in chronic visceral pain, inflammatory pain and bone cancer pain models [64,65,66,67,68]. This wide range of pharmacological activities of minocycline seems to be of key importance in oxaliplatin-induced neuropathic pain, considering that not only inflammatory mechanisms (e.g., increased levels of prostaglandins, IL-1, IL-6, IL-8 and TNF_α_) resulting from alterations in gene expressions (e.g., prostaglandin H_2_ disomerase encoding gene) and the dysregulation of genes associated with neuronal function have been shown to be contributors to oxaliplatin-induced neuropathy and increased neuronal excitability [69].

In recent years, much attention has been focused on the therapeutic potential of minocycline in the neuropathic pain resulting from CIPN. In a mouse model of vincristine-induced neuropathic pain, minocycline prevented the development of mechanical hypersensitivity and the infiltration of immune cells [67]. While minocycline was not fully effective in preventing CIPN caused by paclitaxel, it attenuated acute painful symptoms caused by this taxane derivative [70]. It also reduced oxaliplatin-evoked pain in rats, but was not able to prevent the development of oxaliplatin-induced neuropathic pain [71]. Other studies yielded opposite results. For example, minocycline prevented oxaliplatin-induced mechanical hyperalgesia and intraepidermal nerve fiber loss [72], and the 7 day administration of minocycline (10, 30 mg/kg i.p.) before nerve injury significantly prevented the development of neuropathic pain and delayed the development of hypersensitivity. Interestingly, a single injection of minocycline failed to reverse hypersensitivity when administered during the development of neuropathic pain. No significant effects were observed on hypersensitivity when treatment was introduced in the pre-existing neuropathic pain state. Pretreatment, but not posttreatment with minocycline, markedly attenuated the increased proinflammatory cytokine release and oxidative and nitrosative stress in neuropathic rats. These results suggested that chronic administration of minocycline, when started early before peripheral nerve injury, could attenuate and delay the development of neuropathic pain by inhibiting the release of proinflammatory mediators [73]. Our study also showed that the repeated administration of minocycline 50 and 100 mg/kg to oxaliplatin-treated mice significantly increased the late-phase pre-drug paw withdrawal threshold and prevented the development of late-phase tactile allodynia (Figure 2B). This result proves an important implication of microglial activation in CIPN development.

In our present research, we found that neither the 50 mg/kg minocycline nor 100 mg/kg minocycline used in single-dose or repeated-dose protocols were able to attenuate the cold hyperalgesia caused by oxaliplatin when tested 1 h after intraperitoneal injection (Figure 5A,B), and the effect of the 100 mg/kg minocycline on the cold pain threshold was delayed (Figure 7A). Also, it should be noted here that in the repeated-dose minocycline (100 mg/kg)-treated group, the pre-drug latency to pain reaction in the early phase of the observation was significantly lower than that in the late phase. Such differences were not observed either in the oxaliplatin-treated control mice or in the mice treated with single-dose minocycline (50 mg/kg), repeated-dose minocycline (50 mg/kg), or single-dose minocycline (100 mg/kg) (Figure 5A,B), which indicated that only repeated administrations of minocycline (100 mg/kg) partially prevented the development of late-phase cold hyperalgesia in oxaliplatin-exposed mice.

In the present study, we also investigated whether the antiallodynic and antihyperalgesic properties of minocycline might be enhanced by drugs that act at different targets than minocycline; for this purpose, we focused on duloxetine and ambroxol.

Low-dose (10 mg/kg) intraperitoneal duloxetine affected only early-phase oxaliplatin-induced tactile allodynia (Figure 2C). In contrast, duloxetine at a dose of 30 mg/kg, used in a single-dose protocol, was able to increase the mechanical nociceptive threshold of oxaliplatin-pretreated mice in both phases of the study. Repeated-dose duloxetine (30 mg/kg) was able to increase the mechanical nociceptive threshold of oxaliplatin-pretreated mice in the early phase of the observation. Compared to the oxaliplatin-treated control mice, single and repeated administrations of duloxetine (10 and 30 mg/kg) inhibited the development of late-phase tactile allodynia in oxaliplatin-treated mice, but it is noteworthy that in the late phase of the present study, the increase in the paw withdrawal threshold observed in duloxetine (30 mg/kg)-treated mice significantly exceeded the values obtained for the control animals not exposed to oxaliplatin (Figure 2D). Since for duloxetine some motor impairments were also shown in the rotarod test (Table 1), this might also suggest that these two phenomena might be strongly related to each other.

In the cold plate test, only single-dose and repeated-dose duloxetine (30 mg/kg) was effective in attenuating the cold-induced early-phase pain reaction (Figure 5C). It should also be emphasized that, similar to the repeated-dose minocycline (100 mg/kg), in mice that received repeated-dose duloxetine (30 mg/kg), the pre-drug latency to pain reaction in the early phase of this study was significantly lower than that in the late phase. Such differences were not observed either in the oxaliplatin-treated control mice or in the mice treated with single-dose, repeated-dose duloxetine (10 mg/kg), or single-dose duloxetine (30 mg/kg). This finding indicates that repeated administrations of duloxetine (30 mg/kg) partially attenuated the development of late-phase cold hyperalgesia in oxaliplatin-exposed mice.

Since no preventive therapies have shown significant clinical efficacy for CIPN, drug repurposing may offer an alternative therapeutic option [7]. Previously, it was shown that ambroxol, a mucolytic agent used for the treatment of various respiratory system diseases, is effective as a repurposed drug in numerous neuropathic pain states in humans [34,74,75,76,77]. Additionally, our previous research confirmed that it might attenuate cold hypersensitivity in mice treated with oxaliplatin [78]. Hence, in the next stage of our research, both ambroxol and duloxetine were combined with minocycline to assess how these drug combinations influence mechanical and cold pain thresholds in oxaliplatin-treated animals.

Combination drug therapy (CDT), with the use of two or more drugs with well-defined mechanisms of action, proven analgesic efficacy and well-established adverse effects, often allows analgesia to be achieved with the use of lower doses of combined drugs compared to monotherapy. To avoid a potential increase in combined high-dose drug toxicity in our present study, we combined minocycline, ambroxol and duloxetine at low doses of 50 mg/kg, 90 mg/kg and 10 mg/kg, respectively.

In the tests performed in both the early and late phases of the study, the addition of ambroxol to minocycline (used in both the single-dose and repeated-dose protocols) did not increase the antiallodynic (Figure 3A,B) or antihyperalgesic (Figure 6A,B) efficacy of minocycline used alone in a single-dose or repeated-dose protocol.

Interesting results were obtained for combined minocycline and duloxetine. In the von Frey test performed during the early phase of the study, the single-dose and repeated-dose combinations of minocycline and duloxetine were less effective than the single-dose and repeated-dose minocycline (100 mg/kg), but they were equally as effective as minocycline (50 mg/kg) used alone in the corresponding protocols (Figure 3C). Importantly, single-dose and repeated-dose combinations of minocycline and duloxetine, in contrast to the respective protocols of the administration of minocycline (50 mg/kg) or duloxetine (10 mg/kg), each used alone, showed significant antiallodynic properties in both phases of the study (Figure 3C,D). This important finding shows the potential usefulness and benefits of using this drug combination in reducing tactile allodynia, which results from the administration of oxaliplatin. We also assessed whether combined minocycline and duloxetine was able to prevent late-phase tactile allodynia in mice. In this respect, the single-dose combination was less effective than the repeated-dose combination, and the repeated-dose combination of minocycline and duloxetine was equally as effective as the repeated-dose minocycline (50 mg/kg) used alone.

Additionally, combined minocycline and duloxetine used interventionally in single-dose or repeated-dose protocols were ineffective in reducing cold-induced pain responses in pre-existing neuropathy, either in the early or late phase of the study. Importantly, as in the von Frey test, in the cold plate test we observed that the repeated-dose combination of minocycline and duloxetine inhibited the development of late-phase cold hyperalgesia in oxaliplatin-treated mice. This finding confirms that these combined drugs are superior to minocycline or duloxetine when used alone at the same doses. The mechanism through which both drugs reinforce the other’s antiallodynic and antihyperalgesic effects is not entirely clear, but it can be hypothesized that the above-mentioned multitarget activity of both minocycline and duloxetine might play a key role here.

The beneficial effects of minocycline coadministered with other drugs were also shown previously in a mouse model of CIPN induced by paclitaxel [20]. In this study, the treatment of female mice with indomethacin or minocycline alone did not affect the established paclitaxel-induced thermal hyperalgesia, whereas coadministration of these two drugs attenuated it. Additionally, in male mice, minocycline used alone did not possess antihyperalgesic properties, but the coadministration of both of the drugs had supra-additive antihyperalgesic activity [20]. Consistent with this result, a previous study carried out in a chronic constriction injury model showed that a fixed dose of minocycline (50 mg/kg) combined with ceftriaxone, administered for 7 consecutive days, yielded a potentiating effect and prolonged latency to a noxious thermal stimulus until the 14th day postsurgery, while none of the administered doses of minocycline used alone (25, 50 and 100 mg/kg) and administered intraperitoneally for seven consecutive days from the day of surgery affected thermal hyperalgesia in neuropathic rats. These results suggest that the modulation of microglial activity could have a supportive role in the improvement of thermal hyperalgesia in neuropathic pain, and that the combination of both drugs could be a new and promising therapeutic strategy for neuropathic pain [78].

In our present study, we also assessed the duration of the effect of minocycline and duloxetine on tactile allodynia (Figure 4) and cold hyperalgesia (Figure 7). Minocycline reduced tactile allodynia until 12 h after intraperitoneal injection, and this long-lasting effect was noted either only in the early phase (for minocycline 50 mg/kg, Figure 4A) or in both phases of the study (for minocycline 100 mg/kg, Figure 4A,B). In the von Frey test, the antiallodynic effect of duloxetine at both doses was noted only 1 h after its administration and was limited to the early phase of the observation (Figure 4C,D). In turn, in the cold plate test, minocycline (50 mg/kg) used alone did not possess antihyperalgesic properties 1–12 h after administration (Figure 7A,B), but importantly, minocycline (100 mg/kg), similar to the activity noted in the von Frey test, showed antihyperalgesic properties in the cold plate test during the early phase of the study 6 h after intraperitoneal administration (Figure 7A).

Duloxetine (10 mg/kg) did not show antihyperalgesic properties in the cold plate test 1–12 h after administration (Figure 7C,D); however, the 30 mg/kg dose, similar to the effect observed in the von Frey test, increased the cold pain threshold during the early phase of the observation, 1 h after intraperitoneal injection (Figure 7C).

Considering this finding, we propose combination drug therapy based on the sequential use of duloxetine, a drug with a rapid onset of action, and minocycline, a drug with 12 h antiallodynic and antihyperalgesic activities, as an approach to relieve tactile allodynia and cold hyperalgesia that occur in the course of oxaliplatin-induced neuropathic pain.

We also investigated the impact of the test drugs, used alone or in combination, on the motor skills of mice. In addition to sensory hypersensitivity, motor deficits are a hallmark of CIPN [1]. Therefore, it is of key importance to assess how drugs used to attenuate painful symptoms of neuropathy can influence motor coordination in neuropathic animals. Moreover, tests assessing motor skills enable the exclusion of potential false positive results obtained in pain tests [64].

In the rotarod test, oxaliplatin-treated control mice, compared to control mice not treated with oxaliplatin, showed no motor deficits, confirming that oxaliplatin at the dose that induced sensory deficits did not negatively influence the motor coordination of the mice. The data showing motor deficits in mice treated with duloxetine are consistent with recently published studies [79]. These motor deficits were attenuated when duloxetine was combined with minocycline, which confirms that this drug combination, in contrast to duloxetine used alone, does not have a negative impact on the motor functions in oxaliplatin-treated mice. Of note, in our study, neither minocycline alone nor minocycline combined with duloxetine or ambroxol induced motor impairments in neuropathic oxaliplatin-treated mice. This finding coincides with previously reported data showing that minocycline (100 mg/kg) did not impair the motor activity of mice in the rotarod test [64]. Additionally, earlier studies revealed that combined minocycline and indomethacin showed efficacy in mouse models of CIPN without inducing motor impairments [80].

## 4. Materials and Methods

### 4.1. Animals and Housing Conditions

The behavioral experiments were carried out at the Department of Pharmacodynamics, Faculty of Pharmacy, Jagiellonian University Medical College, Krakow. All tests were performed between 9 a.m. and 2 p.m.. All experimental in vivo procedures were approved by the 1st Local Ethics Committee in Krakow (245/2019, release date: 27 March 2019). The treatment of animals was in full accordance with the ethical standards included in the respective Polish and EU regulations (directive 2010/63/EU). To avoid potential bias in data recording, the investigators involved in the behavioral assays were blinded to the experimental groups. Adult male albino Swiss (CD-1) mice weighing 18–22 g were supplied by the Animal Breeding Farm of the Jagiellonian University Faculty of Pharmacy. Before the in vivo tests, the mice were kept in groups of 10 in standard plastic cages. Bedding material (Transwiór, Poland) was at least 2 cm deep to allow the mice to dig, and the animals were housed under controlled laboratory conditions (room temperature of 22 ± 2 °C, light/dark (12:12) cycle, lights on at 8 a.m., humidity 50 ± 10%, free access to food (Murigran, Agropol, Poland) and tap water). Experimental groups consisted of 6–10 animals/dose. For the behavioral tests, the mice were selected randomly, but the same animals were used throughout the two experimental sessions (i.e., the early phase and the late phase of observation). Between these two sessions, the mice were kept in standard laboratory conditions. After completing the assays on day 7, the mice were euthanized by cervical dislocation.

### 4.2. Chemicals and Drug Administration Scheme

For the in vivo tests, minocycline (Sigma-Aldrich, Poland), duloxetine (donated from Adamed Technology, Poland) and ambroxol (PharmaSwiss/Valeant, Poland) were suspended in 1% Tween 80 (Baxter, Poland). We used the intraperitoneal route for drug administration because the intraperitoneal administration of duloxetine and ambroxol was also used in our present research [41,81]. This approach would enable to compare the results obtained in this present study with the previous ones. Moreover, the intraperitoneal adminstration of test substances is a convenient and widely used method for animal research and, importantly, the pharmacokinetics of substances administered intraperitoneally resembles that seen after oral administration [41]. This is particularly relevant as in humans minocycline, duloxetine and ambroxol are administered orally. We tested antiallodynic and antihyperalgesic properties of these drugs 1 h after their injection because for these substances the time point at which maximal concentration is reached (Tmax) is 1–2.5 h for ambroxol [82], 1.5 h for duloxetine [83] and 1.9 h for minocycline administered orally [84]. Only experiments described in Section 2.4 and Section 2.7 covered several time points of testing.

Control animals (mice not treated with oxaliplatin) and oxaliplatin-treated mice were injected with an appropriate amount of vehicle (1% Tween 80) 1 h before pain tests. Before the experiments, oxaliplatin (Activate Scientific GmbH, Germany) was dissolved in 5% glucose solution (Polfa Kutno, Poland). The doses of oxaliplatin (10 mg/kg, intraperitoneal injection), minocycline (doses: 50 and 100 mg/kg) and duloxetine (doses: 10 and 30 mg/kg) and ambroxol (90 mg/kg) were chosen on the basis of our previous research [41,81,85] and data available in the literature [20,30,86].

### 4.3. Behavioral Tests

#### 4.3.1. Induction of CIPN

Behavioral tests were performed by a trained individual who was blinded to experimental conditions. Immediately after measuring the baseline paw withdrawal threshold or latency to pain reaction in each mouse (for details, please see Section 4.3.2 and Section 4.3.3), neuropathic pain was induced with the use of a single dose of oxaliplatin. Three hours later, the development of early-phase allodynia and hyperalgesia in oxaliplatin-treated mice was measured, and the pre-drug paw withdrawal threshold or latencies to pain reaction were established for each mouse. Then, test drugs or vehicle were administered, and 1 h later, the post-drug paw withdrawal threshold and latencies to pain reaction were collected; this part of the experiment aimed to establish the effect of treatment on early-phase (acute) pain hypersensitivity induced by oxaliplatin. To assess the effect of treatment on late-phase tactile allodynia and cold hyperalgesia, 7 days after oxaliplatin injection, pre-drug and post-drug paw withdrawal thresholds and latencies to pain reactions were measured similarly to those performed during the early phase of this study (Figure 1A). Mechanical and cold pain thresholds were assessed in all experimental groups at both observation time points.

#### 4.3.2. Assessment of Tactile Allodynia—Von Frey Test

The electronic von Frey unit (Bioseb, France) was used for the assessment of mechanical nociceptive threshold (tactile allodynia) in oxaliplatin-treated mice. This device is supplied with a single flexible filament that applies increasing force (from 0 to 10 g) against the plantar surface of the hind paw of a mouse. In the von Frey test, the paw withdrawal response of animals automatically turns off the stimulus, and the mechanical pressure that evokes this response is recorded. On the day of the experiment, the mice were placed individually in test compartments with a wire mesh bottom and were left there for a 1 h habituation. Subsequently, to obtain baseline values, each mouse was tested 3 times alternately in each hind paw. Then, test drugs or vehicle were administered, and 1 h later, 3 measures were taken and averaged to obtain mean post-drug values for each mouse [87].

#### 4.3.3. Assessment of Cold Hyperalgesia—Cold Plate Test

A hot/cold plate apparatus (Bioseb, France) set at 2.5 °C was used to assess the effect of treatment on cold hyperalgesia in oxaliplatin-treated mice. In the cold plate test, the animals were placed on a cold plate, and baseline latencies to pain reactions (i.e., lifting, biting, shaking of hind paws, jumping, movement deficits or writhing response) were measured. These latencies were referred to as ‘before oxaliplatin’ latencies. Then, oxaliplatin was administered, and pre-drug latencies to pain reactions were collected. Finally, test drugs or vehicle were injected, and 60 min later, post-drug latencies to pain reactions were measured. In this assay, a cutoff time of 60 s was established to avoid potential thermally induced damage to paw tissues, and animals not responding within 60 s were removed from the apparatus and assigned a score of 60 s [85,88].

#### 4.3.4. Assessment of Motor Coordination—Rotarod Test

Before the rotarod test, each mouse was subjected to 3 days of training on the rotarod apparatus (Rotarod apparatus, May Commat RR0711, Turkey; rod diameter: 2 cm) that was rotating at a fixed speed of 18 rotations per minute (rpm). During this training session, the mice were placed on the rotating rod for 3 min with an unlimited number of trials. The recorded test was performed 24 h after the last training session. Sixty minutes after the administration of the test drugs or vehicle, the mice were tested on the rotarod that revolved at 6, 18 and 24 rpm (Figure 1B). Motor deficits in mice were defined as the inability to remain on the rotarod apparatus for 1 min. The results are expressed as the mean time spent on the rotarod [87].

### 4.4. Data Analysis

Data analysis was performed using GraphPad Prism software (version 8.0, San Diego, CA, US). Numerical results obtained in behavioral tests are expressed as the mean ± SEM. Statistical analysis was carried out by one-way analysis of variance (ANOVA), followed by Tukey’s post-hoc comparison to compare drug-treated groups vs. the control group. Repeated measures ANOVA and Tukey’s multiple comparison were used for group comparisons made repeatedly at different time points.

## 5. Conclusions

Since many causal mechanisms of CIPN occur simultaneously and can reinforce each other [1], we conclude that both minocycline in monotherapy as well as combination drug therapy based on minocycline and duloxetine may attenuate the symptoms of CIPN. The present study showed that repeated administrations of minocycline (100 mg/kg) and repeated-dose combined minocycline (50 mg/kg) + duloxetine (10 mg/kg) attenuated the development of late-phase tactile allodynia and cold hyperalgesia in oxaliplatin-treated mice, without inducing motor deficits in neuropathic mice. We also propose combination drug therapy based on the sequential use of duloxetine, a drug with a rapid onset of action, and minocycline, a drug with 12 h antiallodynic activity, as an approach to relieve tactile allodynia and cold hyperalgesia that occur in the course of oxaliplatin-induced neuropathic pain.

It should be, however, emphasized that there are some limitations of the present study. Firstly, as reported in the literature [88], in the recent years the lack of translational progress regarding findings from preclinical research to human studies has been noted and not all analgesic active compounds selected with the use of rodent models of CIPN turned out to be effective in humans [89]. Therefore, further studies, including clinical trials assessing the efficacy of minocycline alone or combined with duloxetine, should be carried out to establish whether the results obtained in the present experiment can be translated to patients on oxaliplatin therapy who suffer from CIPN. Secondly, another limitation is that we used a model based on single-dose oxaliplatin. Moreover, the dose range used in the animal model of CIPN and the mode of drug delivery differ from those used in patients treated with oxaliplatin [88,89]. This might also be a potential limitation of the obtained results, leading to a translational failure. Thirdly, in our study we used only male mice and we did not test drugs in female mice. Sex, genotype, neurochemical differences between humans and animals, and affective components of pain and pain-related social communication are key factors that influence pain-associated phenomena [90,91,92]. Not all these issues can be measured in the mouse model used here. Also, it should be noted that this model of neuropathic pain is a manifestation of gain in sensory functions as it measures tactile allodynia and thermal hyperalgesia, whereas many patients suffering from chronic CIPN develop other symptoms, e.g., numbness, ongoing pain and tingling. Fourthly, we used cancer-free mice, whereas in clinical conditions CIPN patients experience cancer, which has a significant impact on the pharmacokinetic and pharmacodynamic properties of drugs. This issue may also compromise the translationality of the results obtained. At the end, one must realize that our in vivo study was focused on the behavioral signs of the adverse effects caused by oxaliplatin, and the pharmacological effects of the drugs tested. We did not investigate if the test compounds influenced the neurophysiological and neuromorphological aspects of CIPN caused by oxaliplatin, and we did not assess the potential adverse drug–drug interactions resulting from the combination drug therapy.

## Figures and Tables

**Table 1:** Effect of test drugs administered in a single-dose or repeated-dose protocol and used alone or in combination on motor coordination of oxaliplatin-treated mice measured in the rotarod test.

Treatment (Drug, Dose, Protocol)	Mean Time [s] (±SEM) on the Rotarod Revolving at:		
	6 rpm	18 rpm	24 rpm
Veh	60.0 ± 0.0	60.0 ± 0.0	47.0 ± 8.5
Veh + oxa	60.0 ± 0.0	60.0 ± 0.0	60.0 ± 0.0
Oxa + Mino 50 single day 1	54.8 ± 5.3	60.0 ± 0.0	53.0 ± 7.0
Oxa + Mino 50 single day 7	60.0 ± 0.0	60.0 ± 0.0	60.0 ± 0.0
Oxa + Mino 50 repeated day 7	60.0 ± 0.0	60.0 ± 0.0	60.0 ± 0.0
Oxa + Mino 100 single day 1	46.8 ± 8.7	48.3 ± 7.7	36.5 ± 9.1
Oxa + Mino 100 single day 7	46.0 ± 5.6	43.8 ± 10.2	42.7 ± 9.6
Oxa + Mino 100 repeated day 7	51.6 ± 5.5	60.0 ± 0.0	55.8 ± 2.8
Oxa + Mino 50 + Am 90 single day 1	57.5 ± 2.5	60.0 ± 0.0	58.3 ± 1.8
Oxa + Mino 50 + Am 90 single day 7	60.0 ± 0.0	60.0 ± 0.0	60.0 ± 0.0
Oxa + Mino 50 + Am 90 repeated day 7	60.0 ± 0.0	60.0 ± 0.0	60.0 ± 0.0
Oxa + Dulo 10 single day 1	60.0 ± 0.0	49.6 ± 5.2	54.3 ± 3.8
Oxa + Dulo 10 single day 7	52.9 ± 4.7	16.8 ± 3.5 ****	9.6 ± 1.7 ****
Oxa + Dulo 10 repeated day 7	60.0 ± 0.0	56.5 ± 2.3	56.0 ± 2.6
Oxa + Dulo 30 single day 1	49.3 ± 7.0	36.0 ± 6.8 **	23.5 ± 8.3 ***
Oxa + Dulo 30 single day 7	44.7 2 6.0	49.5 ± 5.6	16.0 ± 2.8 ****
Oxa + Dulo 30 repeated day 7	54.8 ± 3.4	60.0 ± 0.0	28.8 ± 8.2 **
Oxa + Mino 50 + Dulo 10 single day 1	60.0 ± 0.0	49.8 ± 6.7	57.4 ± 1.7
Oxa + Mino 50 + Dulo 10 single day 7	60.0 ± 0.0	60.0 ± 0.0	48.9 ± 7.3
Oxa + Mino 50 + Dulo 10 repeated day 7	60.0 ± 0.0	49.4 ± 7.0	47.8 ± 7.2

The results are shown as the mean time [s] (± SEM) spent on the rotarod apparatus revolving at 6, 18, and 24 rpm for *n* = 6–8. Statistical analysis: one-way ANOVA followed by Tukey’s post-hoc comparison. Significance vs. vehicle + oxaliplatin-treated mice: ** *p* < 0.01, *** *p* < 0.001, **** *p* < 0.0001. Abbreviations: Veh—vehicle, Oxa—oxaliplatin, Mino—minocycline, Am—ambroxol, Dulo—duloxetine.

## Data Availability

The data presented in this study are available on request from the corresponding author.

## Supplementary Materials

The following are available online. Table S1: Effect of minocycline, duloxetine and ambroxol on tactile allodynia in the oxaliplatin-induced neuropathic pain model, Table S2: Duration of the antiallodynic effect of single-dose minocycline and single-dose duloxetine on the pain threshold in oxaliplatin-treated mice, Table S3: Effect of minocycline, duloxetine and ambroxol on cold hyperalgesia in the oxaliplatin-induced neuropathic pain model, Table S4: Duration of the antihyperalgesic effect of single-dose minocycline and duloxetine, each used alone, on the pain threshold in oxaliplatin-treated mice.

## Abbreviations

Ca_v_	Voltage-gated calcium channel
CDT	Combination drug therapy
CIPN	Chemotherapy-induced peripheral neuropathy
Dulo	Duloxetine
Mino	Minocycline
Na_v_	Voltage-gated sodium channel
Oxa	Oxaliplatin
Veh	Vehicle
