# Supplementary material for: The Microglial Activation Inhibitor Minocycline, Used Alone and in Combination with Duloxetine, Attenuates Pain Caused by Oxaliplatin in Mice
Source: Molecules. 2021 Jun 11;26(12):3577. doi: 10.3390/molecules26123577 (PMC8230860; doi:10.3390/molecules26123577)
Supplement: Supplementary file 1 [file molecules-26-03577-s001.zip › molecules-1239475-supplementary.pdf]

Article

The Microglial Activation Inhibitor Minocycline, Used Alone and in Combination with Duloxetine, Attenuates Pain Caused by Oxaliplatin in Mice

Kinga Sałat [1,*], Anna Furgała-Wojas [1] and Robert Sałat [2]

[1] Department of Pharmacodynamics, Chair of Pharmacodynamics, Faculty of Pharmacy, Jagiellonian University Medical College, 9 Medyczna St., 30-688 Krakow, Poland; anna.furgala@student.uj.edu.pl
[2] Faculty of Electrical and Computer Engineering, Cracow University of Technology, 24 Warszawska St., 31-155 Krakow, Poland; robert.salat@pk.edu.pl
* Correspondence: kinga.salat@uj.edu.pl; Tel/fax: +48 12 620 55 54

Table S1. Effect of minocycline, duloxetine and ambroxol on tactile allodynia in the oxaliplatin-induced neuropathic pain model.

Treatment	Dose [mg/kg]	Protocol	Paw withdrawal threshold [g] ± SEM (early phase)			Paw withdrawal threshold [g] ± SEM (late phase)	
			Before oxa	Predrug	Postdrug	Predrug	Postdrug
Veh	-	Single-dose	2.85±0.09	2.73±0.06	2.8±0.05	2.85±0.08	2.77±0.06
Veh + oxa	-		2.77±0.04	1.75±0.03<br>####	1.8±0.03	1.78±0.04<br>####	1.73±0.03
Oxa + Mino	50		2.87±0.02	1.74±0.05<br>####	1.99±0.05<br>**	1.92±0.06<br>####	2.1±0.06
	100		2.8±0.05	1.71±0.03<br>####	2.19±0.03<br>***	1.89±0.03<br>####	2.27±0.02<br>***
Veh	-		2.85±0.09	2.73±0.06	2.8±0.05	2.85±0.08	2.77±0.06
Veh + oxa	-		2.77±0.04	1.75±0.03<br>####	1.8±0.03	1.78±0.04<br>####	1.73±0.03
Oxa + Dulo	10		2.99±0.08	1.72±0.04<br>####	2.42±0.06<br>****	2.4±0.1<br>#	2.41±0.08
	30		2.78±0.04	1.61±0.03<br>####	3.35±0.03<br>***	2.96±0.05	3.36±0.08<br>***
Veh	-		2.85±0.09	2.73±0.06	2.8±0.05	2.85±0.08	2.77±0.06
Veh + oxa	-		2.77±0.04	1.75±0.03<br>####	1.8±0.03	1.78±0.04<br>####	1.73±0.03
Oxa + Mino + Am	50 (Mino) + 90 (Am)		2.99±0.07	1.72±0.05<br>####	2.06±0.08<br>*	2.11±0.06<br>####	2.23±0.1
Veh	-		2.85±0.09	2.73±0.06	2.8±0.05	2.85±0.08	2.77±0.06
Veh + oxa	-		2.77±0.04	1.75±0.03<br>####	1.8±0.03	1.78±0.04<br>####	1.73±0.03

Oxa + Mino + Dulo	50 (Mino) + 10 (Dulo)		2.73±0.05	1.68±0.04<br>####	1.92±0.03<br>**	1.82±0.03<br>####	1.97±0.02<br>*
Veh	-	Repeated-dose	2.85±0.09	2.73±0.06	2.8±0.05	2.85±0.08	2.77±0.06
Veh + oxa	-		2.77±0.04	1.75±0.03<br>####	1.8±0.03	1.78±0.04<br>####	1.73±0.03
Oxa + Mino	50		2.71±0.08	1.7±0.03<br>####	1.97±0.04<br>**	2.47±0.08	2.5±0.06
	100		2.71±0.06	1.76±0.04<br>####	2.29±0.06<br>***	2.69±0.04	2.99±0.03<br>***
Veh	-		2.85±0.09	2.73±0.06	2.8±0.05	2.85±0.08	2.77±0.06
Veh + oxa	-		2.77±0.04	1.75±0.03<br>####	1.8±0.03	1.78±0.04<br>####	1.73±0.03
Oxa + Dulo	10		2.73±0.05	1.75±0.04<br>####	2.02±0.06	3.11±0.07<br>##	3.29±0.1
	30		2.75±0.07	1.75±0.04<br>####	2.51±0.1<br>***	3.95±0.07<br>####	4.21±0.11
Veh	-		2.85±0.09	2.73±0.06	2.8±0.05	2.85±0.08	2.77±0.06
Veh + oxa	-		2.77±0.04	1.75±0.03<br>####	1.8±0.03	1.78±0.04<br>####	1.73±0.03
Oxa + Mino + Am	50 (Mino) + 90 (Am)		2.78±0.04	1.67±0.04<br>####	1.99±0.07<br>*	2.49±0.06<br>#	2.59±0.05
Veh	-		2.85±0.09	2.73±0.06	2.8±0.05	2.85±0.08	2.77±0.06
Veh + oxa	-		2.77±0.04	1.75±0.03<br>####	1.8±0.03	1.78±0.04<br>####	1.73±0.03
Oxa + Mino + Dulo	50 (Mino) + 10 (Dulo)		2.75±0.07	1.74±0.04<br>####	1.99±0.02<br>***	2.33±0.05<br>###	2.43±0.06<br>*

Effect of test drugs used as single-dose and repeated-dose protocols on pain threshold in oxaliplatin-treated mice was measured in the von Frey test. The results are shown as the mean paw withdrawal threshold [g] ± SEM for n = 9-10. Statistical analysis: repeated measures ANOVA followed by Tukey's post-hoc comparison. Significance: # $p < 0.05$, ### $p < 0.001$, #### $p < 0.0001$ vs. before oxaliplatin; * $p < 0.05$, ** $p < 0.01$, *** $p < 0.001$ vs. pre-drug paw withdrawal threshold in the individual group. In the table 'early phase' and 'late phase' mean two time points at which the measurements were taken (day of oxaliplatin administration and day 7 after a single-dose oxaliplatin administration, respectively). Abbreviations: Veh—vehicle, Oxa—oxaliplatin, Mino—minocycline, Am—ambroxol, Dulo—duloxetine.

Table S2. Duration of the antiallodynic effect of single-dose minocycline and single-dose duloxetine on the pain threshold in oxaliplatin-treated mice.

**Treatment**	**Dose [mg/kg]**	**Time point of testing**	**Time after drug administration**							
			**Paw withdrawal threshold [g] ± SEM (early phase)**				**Paw withdrawal threshold [g] ± SEM (late phase)**			
			1 h	4 h	6 h	12 h	1 h	4 h	6 h	12 h
Oxa + Mino	50	Before oxa	2.87 ±0.02	2.92 ±0.05	2.92 ±0.05	2.91 ±0.05	-	-	-	-
		Predrug	1.74 ±0.05 ####	1.74 ±0.05 ####	1.80 ±0.06 ####	1.78±0.03 ####	1.92 ±0.06 ####	1.77 ±0.03 ####	1.89 ±0.03 ####	1.96 ±0.04 ###
		Postdrug	1.99 ±0.05 **	1.88 ±0.05	2.01 ±0.07 ***	2.07 ±0.06 ***	2.1 ±0.06	1.88 ±0.02	1.99 ±0.05	1.96 ±0.04
	100	Before oxa	2.80 ±0.05	2.91 ±0.06	2.95 ±0.04	3.03 ±0.04	-	-	-	-
		Predrug	1.71 ±0.03 ####	1.74 ±0.05 ####	1.76 ±0.06 ####	1.75 ±0.03 ####	1.89 ±0.03 ####	2.03 ±0.04 ####	2.05 ±0.05 ####	1.94 ±0.05 ####
		Postdrug	2.19 ±0.03 ***	2.29 ±0.09 ***	2.38 ±0.05 ****	2.11 ±0.05 ***	2.27 ±0.02 ***	2.39 ±0.1 ***	2.22 ±0.07	2.25 ±0.05 ***
Oxa + Dulo	10	Before oxa	2.99 ±0.08	2.75 ±0.07	3.36 ±0.02	3.19 ±0.18	-	-	-	-
		Predrug	1.72 ±0.04 ####	1.71 ±0.05 ####	1.73 ±0.05 ####	1.68 ±0.02 ####	2.40 ±0.1 ##	1.91 ±0.06 ###	1.97 ±0.04 ####	1.84 ±0.06 ####
		Postdrug	2.42 ±0.06 ***	1.78 ±0.05	1.94 ±0.03	1.86 ±0.07	2.43 ±0.08	2.06 ±0.03	2.04 ±0.05	2.43 ±0.51 **
	30	Before oxa	2.78 ±0.04	2.78 ±0.04	3.21 ±0.16	3.07 ±0.18	-	-	-	-
		Predrug	1.61 ±0.03 ####	1.73 ±0.04 ####	1.73 ±0.06 ####	1.68 ±0.04 ####	2.96 ±0.05	2.06 ±0.06 ###	2.19 ±0.07 ####	1.97 ±0.06 ####
		Postdrug	3.35 ±0.03 ****	1.83 ±0.04	2.08 ±0.07	2.03 ±0.06	3.36 ±0.08	2.25 ±0.06	2.34 ±0.07	2.14 ±0.06

Effect of minocycline and duloxetine on tactile allodynia measured in the von Frey test assessed 1 h, 4 h, 6 h and 12 h after administration. The test was carried out on the day of oxaliplatin administration (early phase) and 7 days after oxaliplatin (late phase). The results are shown as the mean paw withdrawal threshold [g] ± SEM for n = 10. Statistical analysis: repeated measures ANOVA followed by Tukey's post-hoc comparison. Significance: ## $p < 0.01$, ### $p < 0.001$, #### $p < 0.0001$ vs. before oxaliplatin; ** $p < 0.01$, *** $p < 0.001$, **** $p < 0.0001$ vs. pre-drug paw withdrawal threshold. Abbreviations: Oxa—oxaliplatin, Mino—minocycline, Dulo—duloxetine.

Table S3. Effect of minocycline, duloxetine and ambroxol on cold hyperalgesia in the oxaliplatin-induced neuropathic pain model.

Treatment	Dose [mg/kg]	Protocol	Latency to pain reaction [s] (± SEM) (early phase)			Latency to pain reaction [s] (± SEM) (late phase)	
			Before oxa	Predrug	Postdrug	Predrug	Postdrug
Veh	-	Single-dose	57.6±1.27	59.8±0.16	58.5±0.86	57.6±1.26	58.0±0.63
Veh + oxa	-		48.9±3.72	14.2±5.48 ###	10.1±3.31	18.2±5.60 ###	10.2±3.23
Oxa + Mino	50		59.4±0.53	6.7±1.47 ###	10.1±1.53	12.3±3.54 ###	8.7±1.79
	100		57.1±1.65	14.0±5.63 ###	21.4±6.79	10.1±2.69 ###	12.7±5.58
Veh	-		57.6±1.27	59.8±0.16	58.5±0.86	57.6±1.26	58.0±0.63
Veh + oxa	-		48.9±3.72	14.2±5.48 ###	10.1±3.31	18.2±5.60 ###	10.2±3.23
Oxa + Dulo	10		52.4±2.93	10.2±3.06 ###	17.4±4.35	21.6±5.37 ###	15.0±2.48
	30		57.0±2.50	11.4±2.29 ###	35.7±7.06 *	23.1±6.56 ###	20.2±7.34
Veh	-		57.6±1.27	59.8±0.16	58.5±0.86	57.6±1.26	58.0±0.63
Veh + oxa	-		48.9±3.72	14.2±5.48 ###	10.1±3.31	18.2±5.60 ###	10.2±3.23
Oxa + Mino + Am	50 (Mino) + 90 (Am)		59.1±0.85	8.4±1.70 ####	7.9±1.62	11.46±2.05 ####	8.25±2.39
Veh	-		57.6±1.27	59.8±0.16	58.5±0.86	57.6±1.26	58.0±0.63
Veh + oxa	-		48.9±3.72	14.2±5.48 ###	10.1±3.31	18.2±5.60 ###	10.2±3.23
Oxa + Mino + Dulo	50 (Mino) + 10 (Dulo)		57.0±2.1	11.6±4.82 ####	6.00±1.18	9.4±2.31 ####	7.7±1.79
Veh	-	Repeated-dose	57.6±1.27	59.8±0.16	58.5±0.86	57.6±1.26	58.0±0.63
Veh + oxa	-		48.9±3.72	14.2±5.48 ###	10.1±3.31	18.2±5.60 ###	10.2±3.23
Oxa + Mino	50		54.7±2.49	10.5±1.37 ###	10.6±2.28	15.48±5.23 ###	7.3±1.81
	100		56.1±2.48	9.8±2.48 ###	18.1±5.41	41.2±7.71 ###	27.1±5.55
Veh	-		57.6±1.27	59.8±0.16	58.5±0.86	57.6±1.26	58.0±0.63
Veh + oxa	-		48.9±3.72	14.2±5.48 ###	10.1±3.31	18.2±5.60 ###	10.2±3.23
Oxa + Dulo	10		53.3±2.78	8.1±1.34 ###	11.3±2.63	20.6±4.84 ###	23.9±5.33
	30		56.9±2.16	13.4±6.10 ###	35.1±7.72 *	34.7±6.54 ###	35.2±6.73

Veh	-		57.6±1.27	59.8±0.16	58.5±0.86	57.6±1.26	58.0±0.63
Veh + oxa	-		48.9±3.72	14.2±5.48 ###	10.1±3.31	18.2±5.60 ###	10.2±3.23
Oxa + Mino + Am	50 (Mino) + 90 (Am)		55.0±2.58	13.8±5.59 ###	13.4±5.42	18.2±7.14 ##	13.5±5.64
Veh	-		57.6±1.27	59.8±0.16	58.5±0.86	57.6±1.26	58.0±0.63
Veh + oxa	-		48.9±3.72	14.2±5.48 ###	10.1±3.31	18.2±5.60 ###	10.2±3.23
Oxa + Mino + Dulo	50 (Mino) + 10 (Dulo)		50.8±3.30	7.5±1.58 ####	22.3±6.03	32.6±8.91	22.6±5.48

Effect of test drugs used as single-dose and repeated-dose protocols on pain threshold in oxaliplatin-treated mice was measured in the cold plate test. The results are shown as the mean latency to pain reaction [s] ± SEM for n = 9-10. Statistical analysis: repeated measures ANOVA followed by Tukey's post-hoc comparison. Significance: ## $p < 0.01$, ### $p < 0.001$, #### $p < 0.0001$ vs. before oxaliplatin; * $p < 0.05$ vs. pre-drug paw withdrawal threshold in the individual group. In the table 'early phase' and 'late phase' mean two time points at which the measurements were taken (day of oxaliplatin administration and day 7 after a single-dose oxaliplatin administration, respectively). Abbreviations: Veh—vehicle, Oxa—oxaliplatin, Mino—minocycline, Am—ambroxol, Dulo—duloxetine.

Table S4. Duration of the antihyperalgesic effect of single-dose minocycline and duloxetine, each used alone, on the pain threshold in oxaliplatin-treated mice.

Treatment	Dose [mg/kg]	Time point of testing	Time after drug administration							
			Latency to pain reaction [s] ± SEM (early phase)				Latency to pain reaction [s] ± SEM (late phase)			
			1 h	4 h	6 h	12 h	1 h	4 h	6 h	12 h
Oxa + Mino	50	Before oxa	59.4<br>±0.53	57.4<br>±2.02	55.2<br>±2.37	55.0<br>±2.82	-	-	-	-
		Predrug	6.7<br>±1.47<br>####	9.5<br>±2.76<br>####	14.3<br>±5.55<br>####	13.6<br>±4.24<br>####	12.3<br>±3.54<br>####	8.5<br>±1.92<br>####	15.4<br>±5.54<br>####	15.8<br>±5.25<br>####
		Postdrug	10.1<br>±1.53	10.4<br>±2.15	18.2<br>±6.06	18.3<br>±3.08	8.7<br>±1.79	12.3<br>±3.92	5.7<br>±0.62	13.6<br>±5.36
	100	Before oxa	57.1<br>±1.65	54.2<br>±2.67	48.9<br>±2.74	54.1<br>±2.46	-	-	-	-
		Predrug	14.0<br>±5.63<br>####	16.1<br>±6.23<br>####	9.7<br>±1.85<br>####	16.9<br>±7.03<br>####	10.1<br>±2.69<br>####	18.2<br>±3.85<br>####	12.8<br>±2.51<br>####	8.6<br>±1.54<br>####
		Postdrug	21.4<br>±6.79	15.5<br>±4.38	25.0<br>±5.99<br>*	6.4<br>±1.72	12.7<br>±5.58	8.5<br>±3.37	6.8<br>±2.85	10.6<br>±3.39
Oxa + Dulo	10	Before oxa	52.4<br>±2.93	57.8<br>±2.23	55.4<br>±2.41	57.3<br>±1.8	-	-	-	-
		Predrug	10.2<br>±3.06<br>####	18.1<br>±5.71<br>####	15.8<br>±3.63<br>####	9.7<br>±3.75<br>####	21.6<br>±5.37<br>####	9.4<br>±2.08<br>####	7.7<br>±1.39<br>####	14.5<br>±2.21<br>####
		Postdrug	17.4<br>±4.35	14.3<br>±3.70	18.7<br>±5.71	17.0<br>±3.89	15.0<br>±2.48	7.2<br>±1.83	12.5<br>±3.30	8.1<br>±3.3
	30	Before oxa	57.0<br>±2.50	57.1<br>±2.27	54.3<br>±3.06	53.6<br>±3.29	-	-	-	-
		Predrug	11.4<br>±2.29<br>###	17.0<br>±5.22<br>####	16.3<br>±5.0<br>####	11.2<br>±2.36<br>####	23.1<br>±6.56<br>####	9.1<br>±1.91<br>####	7.6<br>±2.79<br>####	12.3<br>±3.39<br>####
		Postdrug	35.7<br>±7.06<br>***	20.4<br>±7.02	11.1<br>±3.17	16.3<br>±5.83	20.2<br>±7.34	9.3<br>±1.75	9.7<br>±2.36	7.8<br>±2.55

Effect of minocycline and duloxetine on cold hyperalgesia measured in the cold plate test assessed 1 h, 4 h, 6 h and 12 h after administration. The test was carried out on the day of oxaliplatin administration (early phase) and 7 days after oxaliplatin (late phase). The results are shown as the mean latency to pain reaction [s]± SEM for n = 10. Statistical analysis: repeated measures ANOVA followed by Tukey's post-hoc comparison. Significance: ### $p < 0.001$, #### $p < 0.0001$ vs. before oxaliplatin; * $p < 0.05$, *** $p < 0.001$ vs. pre-drug latency to pain reaction. Abbreviations: Oxa—oxaliplatin, Mino—minocycline, Dulo—duloxetine.
